# Which strain of the avian coronavirus vaccine will become the prevalent one in China next?

**DOI:** 10.3389/fvets.2023.1139089

**Published:** 2023-05-05

**Authors:** Haizhu Li, Gengsong Liu, Qiaoyan Zhou, Hongchun Yang, Congcong Zhou, Weili Kong, Jieyu Su, Gonghe Li, Hongbin Si, Changbo Ou

**Affiliations:** ^1^College of Animal Science and Technology, Guangxi University, Nanning, China; ^2^Gladstone Institute of Virology, University of California, San Francisco, San Francisco, CA, United States; ^3^Guangxi Zhuang Autonomous Region Engineering Research Center of Veterinary Biologics, Nanning, China; ^4^Guangxi Key Laboratory of Animal Reproduction, Breeding and Disease Control, Nanning, China

**Keywords:** infectious bronchitis virus, coronavirus, vaccine strain, S1 gene, genotype, Newcastle disease virus, vector vaccine

## Abstract

Infectious bronchitis virus (IBV) is a vital pathogen in poultry farms, which can induce respiratory, nephropathogenic, oviduct, proventriculus, and intestinal diseases. Based on the phylogenetic classification of the full-length S1 gene, IBV isolates have been categorized into nine genotypes comprising 38 lineages. GI (GI-1, GI-2, GI-3, GI-4, GI-5, GI-6, GI-7, GI-13, GI-16, GI-18, GI-19, GI-22, GI-28, and GI-29), GVI-1 and GVII-1 have been reported in China in the past 60 years. In this review, a brief history of IBV in China is described, and the current epidemic strains and licensed IBV vaccine strains, as well as IBV prevention and control strategies, are highlighted. In addition, this article presents unique viewpoints and recommendations for a more effective management of IBV. The recombinant Newcastle Disease virus (NDV) vector vaccine expressed S gene of IBV QX-like and 4/91 strains may be the dominant vaccine strains against NDV and IBV.

## 1. Introduction to animal coronavirus

Since the outbreak of Coronavirus Disease 2019 (COVID-19), there are more than 642 million confirmed cases and 6.6 million confirmed deaths caused by the coronavirus infection by December 7, 2022 in World Health Organization website. Since the first confirmed case was announced, the COVID-19 pandemic has lasted almost 3 years, and the virus has caused significant economic loss throughout the world (1). In order to tackle the virus, the scientists and health workers demonstrated their professionalism. From the initial case care to the current vaccine immunization, an increasing number of nations will consider opening their borders when the vaccination rate exceeds 85%, indicating that vaccine immunization remains an essential technique for preventing coronavirus infection.

Animal coronavirus never leaves our attention, and there are a few important coronaviruses that severely harm the cattle and poultry industries’ economies, such as porcine transmissible gastroenteritis virus (TGEV), porcine epidemic diarrhea virus (PEDV), porcine Delta coronavirus (PDCoV), canine coronavirus (CCoV), feline coronavirus (FCoV) and avian infectious bronchitis virus (IBV) (Table 1). Although the first coronavirus was discovered in the 1930s, it gained widespread attention when the severe acute respiratory syndrome (SARS) epidemic caused by the SARS coronavirus swept throughout numerous countries and areas from 2002 to 2003 and sparked widespread social fear (2). Prior to this, the research on coronavirus was mostly limited to the veterinary field. Avian infectious bronchitis virus is highly contagious in chickens; it is a major cause of serious respiratory disorders and poses a significant threat to the poultry industry, also in China.

**Table 1:** Main animal coronavirus.

Coronavirus	Animal virus	Susceptible animal
alpha	PEDV, TGEV, CCoV, FCoV	Mammal
beta	BCoV, ECoV, PHEV, CrCoV, MHV	
gamma	IBV, BWCoV-SW1	avian
delta	PDCoV, COMCoV	Mammal/avian

BCoV, Bovine coronavirus; ECoV, equine coronavirus; CrCoV, canine respiratory coronavirus; MHV, mouse hepatitis virus; PHEV, porcine hemagglutinating encephalomyelitis virus; BWCoV-SW1, beluga whale coronavirus SW1; CMCoV, common moorhen coronavirus.

IBV belongs to gamma group of coronavirus. Its complete genome is about 27.6 kb and composed of 5′-1a-1b-S(S1, S2)-3a-3b-3c(E)-M-5a-5b-N-Poly(A)-3′ from 5′ end to 3′ end (3). The IBV genome encodes four structure proteins, namely spike protein (S), small envelope protein (E), membrane protein (M) and nucleocapsid protein (N). Among the four structure proteins, S protein contains main protective antigen epitopes of IBV and can stimulate the body to produce neutralizing antibodies. Meanwhile, S protein also plays a vital role in the process of viral adsorption into cells, thus it determines the tissue tropism of the virus to a certain extent (4). A small part of the C end of S protein is buried in the envelope membrane, and the rest is located outside the envelope membrane to form the viral spike. The S protein is composed of S1 at the N-terminal of amino acid and S2 at the C-terminal of amino acid, which are composed of 520 and 625 amino acids, respectively, and are joined by disulfide bonds (5, 6). In addition, the S1 protein has three highly variable regions that are closely associated with IBV serotype, indicating that the S1 gene nucleotide is the primary determinant for IBV genotyping (7).

## 2. History of IBV in China

IB was first described in United States in 1931 as a respiratory disease of chickens (8). In 1965, the world’s first human coronavirus was isolated from the nasal discharge of patients with the common cold (9). In 1975, the coronavirus was officially named as *Coronaviridae* by the International Committee on Nomenclature of Viruses (now it is renamed as the International Committee on Taxonomy of Viruses (ICTV)) and the *Coronaviridae* is currently divided into two genera Coronavirus and Cyclovirus based on the viral serological characteristics and nucleotide sequence differences. The common important coronavirus such as SARSV, IBV, TGEV and PEDV belongs to coronavirus genus.

**Figure 1:**
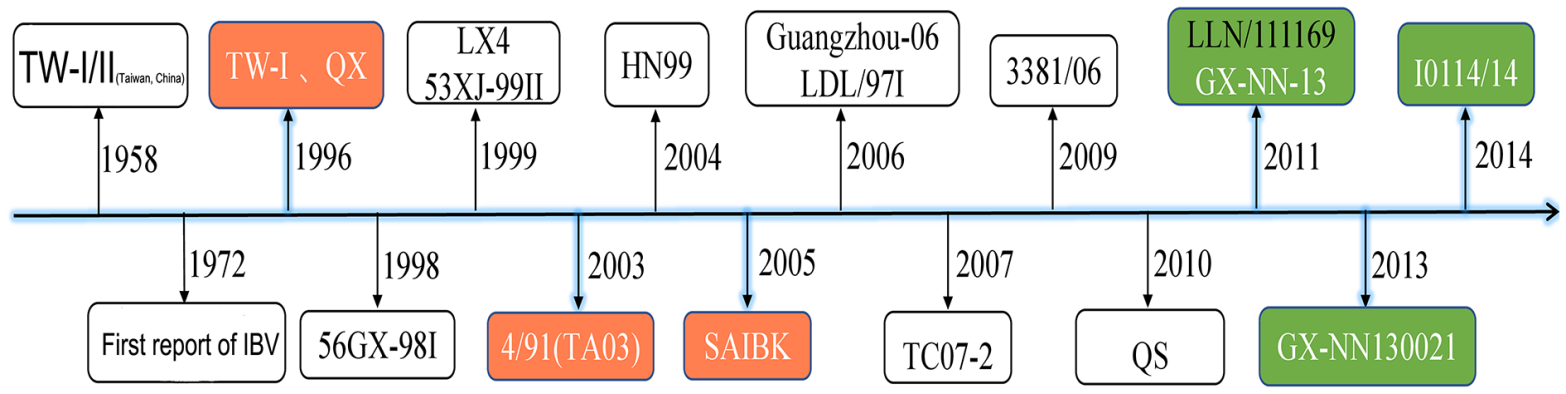
Main representative events in the history of IBV in China.

IBV has existed more than 60 years in China (Figure 1). In 1958, IBV outbreak occurs in Taiwan Province of China. Genotypes TW-I and TW-II were firstly identified in the early 1990s and considered to be of distinct genotypes (10). In 1972, IBV was first reported in a poultry farm of Chinese mainland (11). IBV then rapidly becomes one of the most important pathogens in poultry industry. According to the tissue tropism of IBV, IBV isolation strains were initially classified as nephropathogenic, oviduct, proventriculus, or intestine type (12–15). With the advancement of molecular biology technology, the phylogenetic classification of IBV isolates began to be categorized into nine genotypes consisting of 38 lineages based on the full-length S1 gene, and the genotype has become the most used method for the classification of IBV isolation strains (16–20). The genotype I includes 30 distinct viral lineages (temporarily named GI-1 to GI-30), while each of the remaining genotypes consists of one viral lineage (GII-1, GIII-1 GIV-1, GV-1, GVI-1, GVII-1, GVIII-1, and GIX-1) (20, 21).

**Figure 2:**
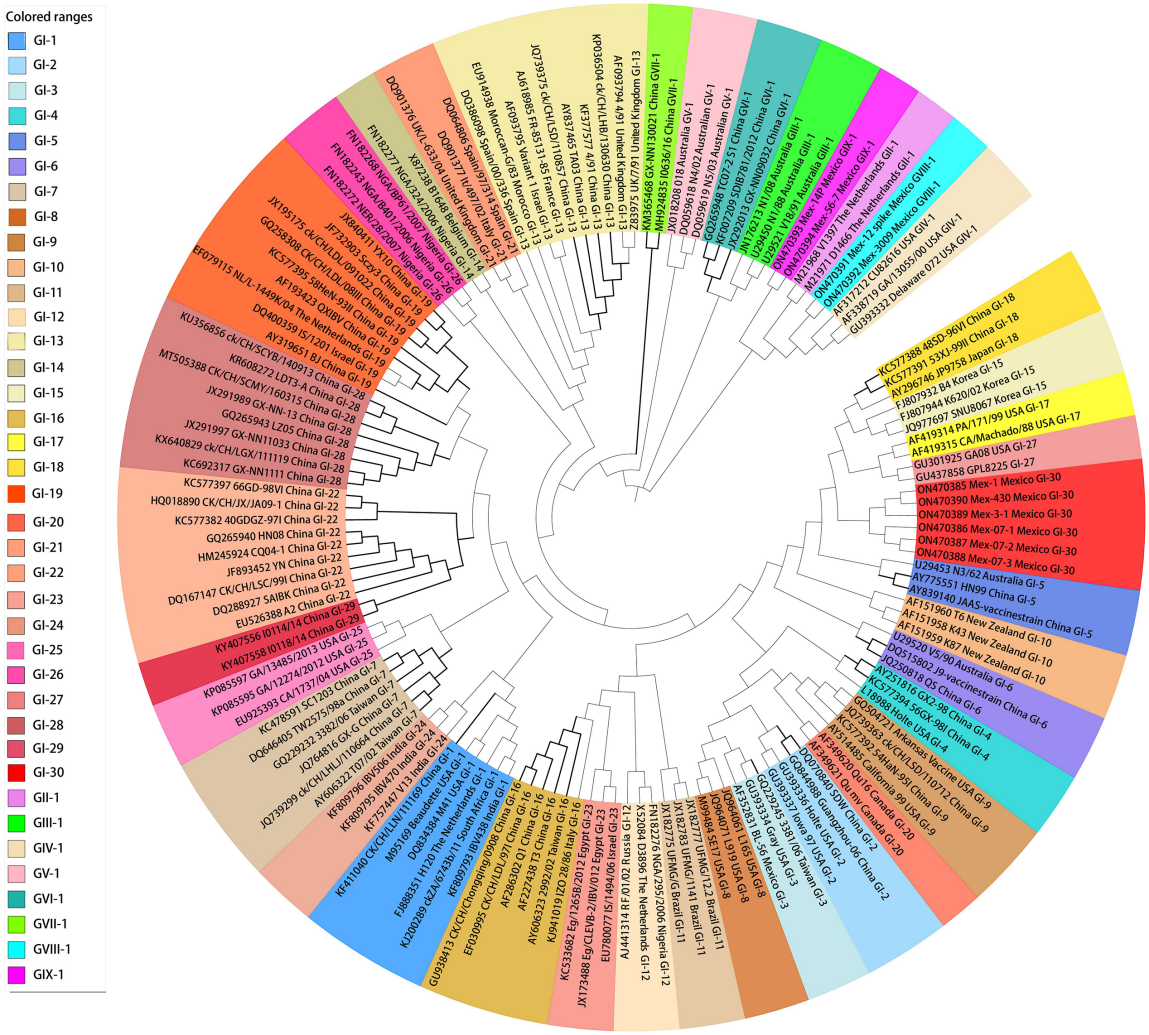
Genetic phylogenetic tree based on the nucleotide sequence analysis of S1 gene of IBV. The tree was constructed using the neighbor-joining method by the MEGA 11.0 software. 143 S1 gene sequences of IBV reference strains were clustered into GI–GIX genotypes with 30 GI lineages. All branch lines of China are highlighted.

Gene mutation and recombination (especially in the *S1* gene) may give rise to variants or new genotypes. Many SARS-CoV-2 variants emergence in the past 3 years told us the rapid mutation rate of all coronaviruses including IBV, which can induce the emergence of new virus types with the potential to cause disease (8, 22). Due to Chinese unique geographical landforms and large differences in climate and environment, as well as different breeding varieties and breeding modes in the north and south part of China, the epidemic characteristics of IBV vary in different times and regions and a variety of IBV genotypes and variants in China (23). GI (GI-1, GI-2, GI-3, GI-4, GI-5, GI-6, GI-7, GI-13, GI-16, GI-18, GI-19, GI-22, GI-28, and GI-29), GVI-1 and GVII-1 have been reported in China in the last few years (18, 24–26). Among these reported viral lineages, the more representative and important genotypes are QX (GI-19) and 4/91 (GI-13), which are the dominant lineages in China. It is reported that 93 QX strains and 19 4/91 strains were isolated in South China from April 2019 to March 2020, which are the first and second epidemic strains in South China, respectively (27). Moreover, TW (GI-7) and SAIBK (GI-22) occasionally cause epidemic in some regions. GI-28, GI-29, and GVII-1 are the new genotypes isolated in recent years (Figure 2).

The glandular stomach type IBV QX strain was first isolated from diseased chicken flocks in Qingdao in 1996, and a representative strain LX4 was isolated from diseased chickens in Xinjiang in 1999, which has high nucleotide similarity with a Korean nephropathogenic isolation strain KM91 (23, 28–30). Later, a number of IBV strains similar to QX-like strains were identified and become the dominant epidemic strains in China (28). Then similar viruses were reported in Eastern Europe in 2001 (31) and other European countries including the United Kingdom (32, 33). Now the QX-like type IBV as a member of the GI-19 group represents one of the most important IBV genotypes globally due to its wide prevalence and distribution and recombination with vaccine strains and field strains (8, 24). A novel low virulent respiratory IBV ck/CH/LJX/2017/07 (referred as JX17) was isolated from chicken vaccinated with H120 and 4/91 and proved to be the recombination strain of QX, TW and 4/91 genotype strains (34). A highly virulent QX-like IBV strain CK/CH/JS/TAHY displayed classical clinical signs including coughing, nasal discharge, and kidney damage with severe urate deposition (35). Novel QX-like IBV strains not only can be isolated from intensive poultry farms with strict immune program, but also display a variety of clinical symptoms, indicating that it is difficult to distinguish variants depending on tissue tropisms.

The first known 4/91 (also known as 793/B and CR88) serotype isolation strain was isolated in France in 1985 and then entered the United Kingdom in the winter of 1990 (8, 36, 37). Subsequently, this serotype was prevalent in the majority of European countries and in many other regions of the world, but its structure and antigenicity do not appear to have altered significantly, since the initially designed vaccines continue to provide effective immunity (38). In 2003, the first 4/91 serotype strain was isolated in China, and the virus isolate was designated Taian-03 (also TA03). Since then, the 4/91 serotype virus strain has been discovered and is gaining prevalence in China (39). More importantly, gene recombination involved by the 4/91 vaccine and field strains result in the emergence of several novel strains and further increase the genetic diversity and complexity of infectious bronchitis virus (18, 40, 41). The novel GI-28 isolation strain CK/CH/SCMY/160315 was found to be a multiple recombinant strain originating from live attenuated vaccine strains H120 (GI-1), 4/91 (GI-13), LDT3-A (GI-28) and the field strain LJL/08–1 (GI-19) (24). Likely, an IBV strain ck/CH/LHLJ/140906 was also a natural recombination strain from parental IBV strains 4/91 and H120 (42). Previous studies have displayed that the 4/91 type strains were more prone to recombination and hence the 4/91 type vaccine should be used sparingly or with caution (41).

TW genotype is an important gene type and the starts to become the most common genotype in mainland of China recently (39, 41). IBV Taiwan strains were divided into TW-I and TW-II genotypes and grouped into the same lineage GI-7 (16). Due to the geographical barrier between Taiwan and mainland of China, the IBV Taiwan strains are evolutionarily distinct from IBV isolation strains of mainland China (43). The TW-I genotype strains were frequently isolated in mainland China after 1990s with classical nephritis symptoms. The TW-II genotype strains characterized with respiratory symptoms were isolated from Taiwan China before 1990s, now it is rare to isolate this genotype virus in mainland China (10, 44). Since 2009, the prevalence of TW-I genotype IBVs has been increasing in mainland China from 7.5% (2009–2010) to 27.4% (2013–2015) (39, 45). Notably, the TW-I genotype was predominant in Guangdong, Guangxi, and Fujian, three southern provinces of China due to selective pressure from extensive vaccination and close distance (39).

Two novel serotypes have been added into Genotype I IBV in China. The IBV strain ck/CH/LGX/111119 was found to be genetically and antigenically different from other known IBV types and designated as GI-28 based on the results of S1 sequence analysis and virus cross-neutralization tests. It is a nephropathogenic strain with a broader tissue tropism and may be a recombination of LX4 genotype (GI-19) and as-yet-unidentified IBVs (17). Then, many GI-28 genotype IBV strains were isolated in different regions of China, such as CK/CH/SCMY/160315 strain and GX-NN-13 (24, 39, 41). Another novel genotype (GI-29) IBV strains, including γCoV/ck/China/I0111/14, γCoV/ck/China/I0114/14 and γCoV/ck/China/I0118/14, were isolated from Guangxi of south China and were three nephropathogenic strains (19).

The GVII-1 genotype was another novel IBV lineage in China. The GX-NN130021 strain was isolated from suspected IBV-positive 21 day-old diseased chickens, which were vaccinated by the H120 vaccine, in Guangxi province of China in 2013 and were designated as new-type I (46). An epidemiological study on genetic diversity of avian IBV from January 2016 to December 2017 in China displayed a new variant I0636/16 which was highly homologous with the GX-NN130021 strain based on sequence analysis of the complete nucleotide sequences of S1 gene (47). Until 2019, both I0636/16 and GX-NN130021 strains were grouped into the GVII-1 genotype based on detailed phylogenetic analysis and sequence comparisons. Meanwhile, the virus cross-neutralization results showed that GVII-1 was antigenically different from the currently genotype lineages (18). The GVII-1 genotype has been circulating in China over a 9-year period.

## 3. Current prevention and control strategy against IBV in China

Currently, vaccination inoculations with live attenuated or inactivated vaccines remain the most effective technique for protecting chickens from IBV infection in poultry farms around the globe. In addition to comprehensive biosafety epidemic prevention measures, it is crucial to develop appropriate immunization protocols to prevent and control the IBV infection. There are numerous types of IBV vaccinations in China, including live and inactivated vaccines as well as single and multiple combined vaccines. Moreover, there are also many types of IBV vaccine strains, such as Mass, QX-like and 4/91-like types.

All available commercial IBV vaccines were summarized in Table 2 based on the National Basic Veterinary Drug Database.^1^ These vaccines exhibit several characteristics. First, all single vaccines are live attenuated IBV vaccine strains and grouped into three types: Mass (H52, H120 and W93), QX-like (LDT3-A and HC13-91), and 4/91-like (NNA and FNO-55), which represents the current main epidemic strains in China. Second, the NDV-IBV combined vaccines are subdivided into live and inactivated vaccine, and the inactivated NDV-IBV combined vaccine has only two kinds of IBV strains M41 (Mass) and Jin 13 (QX-like). There are several genotypes for the live NDV-IBV combined vaccine, including H52, H120, QXL87, B48, and LDT3-A. All triple and quadruple inactivated IBV vaccinations are derived from the same IBV vaccine strain M41. In all, IBV and NDV are usually used to make combined vaccine together.

**Table 2:** Commercial IBV vaccines list in China.

Vaccine type	Vaccine name	Strain type	Number of companies
Binary IBV vaccine	NDV + IBV inactivated vaccine	LaSota+M41	3
	NDV + IBV inactivated vaccine	Ulster2C + M41	1
	NDV + IBV inactivated vaccine	LaSota+Jin 13	3
Triple IBV vaccine	NDV + IBV + AIV inactivated vaccine	LaSota+M41 + WD	6
	NDV + IBV + AIV inactivated vaccine	LaSota+M41 + L	5
	NDV + IBV + AIV inactivated vaccine	LaSota+M41 + LG1	1
	NDV + IBV + AIV inactivated vaccine	LaSota+M41 + SS	4
	NDV + IBV + AIV inactivated vaccine	N7a + M41 + SZ	2
	NDV + IBV + AIV inactivated vaccine	LaSota+M41 + SS/94	2
	NDV + IBV + AIV inactivated vaccine	LaSota+M41 + H9 HL	3
	NDV + IBV + AIV inactivated vaccine	LaSota+M41 + YBF003	1
	NDV + IBV + EDSV inactivated vaccine	LaSota+M41 + Z16	5
	NDV + IBV + EDSV inactivated vaccine	LaSota+M41 + Jing911	1
	NDV + IBV + EDSV inactivated vaccine	LaSota+M41 + HZ	4
	NDV + IBV + EDSV inactivated vaccine	LaSota+M41 + HSH23	6
	NDV + IBV + EDSV inactivated vaccine	LaSota +M41 + AV127	6
	NDV + IBV + EDSV inactivated vaccine	Clone30 + M41 + AV127	3
	NDV + IBV + EDSV inactivated vaccine	LaSota+M41 + K-11	1
	NDV + IBV + EDSV inactivated vaccine	N79 + M41 + NE4	1
	NDV + IBV + AIV inactivated vaccine	LaSota+M41 + H9 HP	4
	NDV + IBV + AIV inactivated vaccine	LaSota+M41 + NJ02	6
	NDV + IBV + IBDV inactivated vaccine	LaSota+M41 + HQ	2
	NDV + IBV + IBDV inactivated vaccine	LaSota+M41 + S-VP2 Protein	2
	NDV + IBV + IBDV inactivated vaccine	Ulster2C + M41 + VNJO	1
	NDV+IBV+EDSV inactivated vaccine	Ulster2C + M41 + 127	1
	NDV + IBV + EDSV inactivated vaccine	LaSota+M41 + HE02	6
	NDV + IBV + AIV inactivated vaccine	LaSota+M41 + Re-9	2
	NDV + IBV + AIV inactivated vaccine	LaSota+M41 + SY	2
	NDV + IBV + AIV inactivated vaccine	LaSota+M41 + HN106	6
Quadruple IBV vaccine	NDV + IBV + AIV + IBDV inactivated vaccine	LaSota+M41 + H9 SZ + rVP2 Protein	9
	NDV + IBV + AIV + IBDV inactivated vaccine	LaSota+M41 + YBF003 + S-VP2 Protein	1
	NDV + IBV + AIV + IBDV inactivated vaccine	N7a + M41 + SZ + rVP2 Protein	2
	NDV + IBV + EDSV + AIV(H9) inactivated vaccine	LaSota +M41 + AV127 + NJ02	12
	NDV + IBV + EDSV+ AIV(H9) inactivated vaccine	LaSota +M41 + AV127 + HL	2
	NDV + IBV + EDSV+ AIV(H9) inactivated vaccine	LaSota +M41 + NE4 + YBF003	1
	NDV + IBV + EDSV+ IBDV inactivated vaccine	Ulster2C + M41 + 127 + VNJO	1
	NDV + IBV + IBDV+ Viral Arthritis inactivated vaccine	LaSota+M41 + S-VP2 Protein+AV2311	1
	NDV + IBV + EDSV+ IBDV inactivated vaccine	LaSota+M41 + Z16 + HQ	2
	NDV + IBV + EDSV + AIV inactivated vaccine	LaSota+M41 + Z16 + HP	5
	NDV + IBV + EDSV + AIV(H9) inactivated vaccine	LaSota+M41 + HS25 + HZ	4
	NDV + IBV + EDSV + AIV inactivated vaccine	LaSota+M41 + HE02 + HN106	3
	NDV + IBV + EDSV+ AIV inactivated vaccine	LaSota+M41 + HSH23 + WD	8
	NDV + IBV + EDSV+ AIV inactivated vaccine	LaSota+M41 + K-11 + SS/94	1
	NDV + IBV + EDSV+ AIV inactivated vaccine	LaSota+M41 + AV-127 + S2	1
	NDV + IBV + EDSV+ AEV inactivated vaccine	LaSota+M41 + HSH23 + Van Roekel	3

NDV, newcastle disease virus; IBDV, infectious bursal disease virus; EDSV, egg drop syndrome virus; AEV, avian encephalomyelitis virus.

Although there are numerous vaccines available, there are numerous predominant IBV serotype strains that compromise the vaccine’s effectiveness. Different IBV serotypes only induce limited cross-protective immunity between different serotypes, thus vaccination immunization failures are often reported. Therefore, the key to preventing IBV infection is timely knowledge of the precise local epidemic strain and selection of the appropriate IBV serotype vaccination.

**Figure 3:**
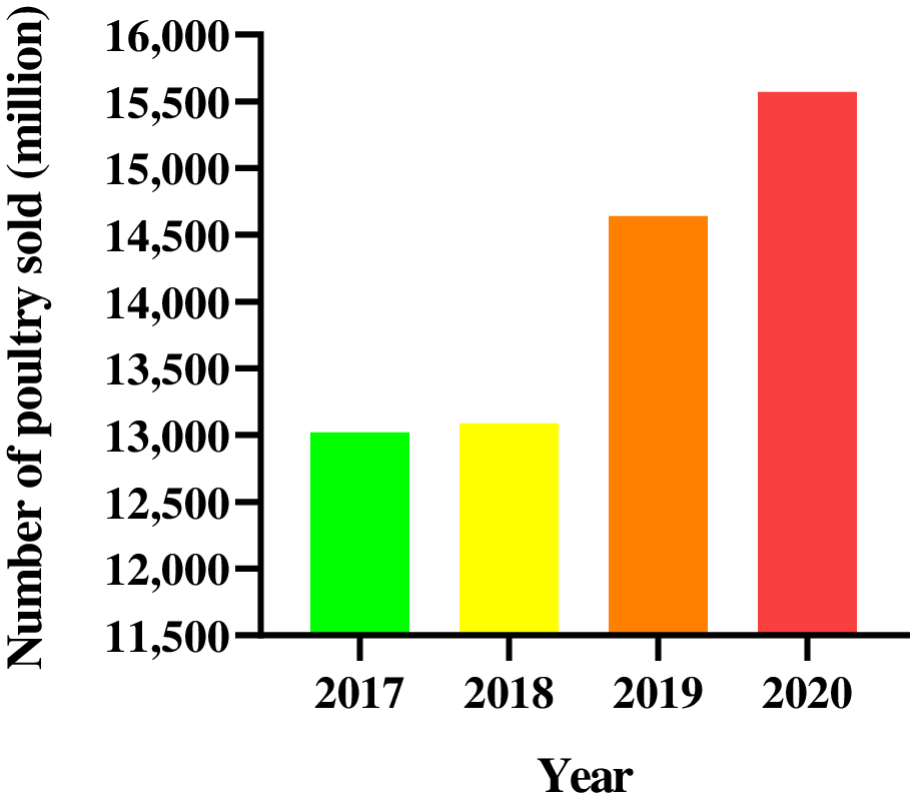
Number of chickens marketed in China from 2017 to 2020.

In recent years, the number of chickens marketed in China has risen significantly from 2017 to 2020, reaching 15 billion by the end of 2020 (Figure 3). IBV outbreak occasionally occurs in large-scale farms and scattering raising-households. To combat with IBV immunization failure certain strategies for the control of virus infection can be implemented. Traditional Chinese medical sciences (especially acupuncture) have been verified by recent studies (48) and traditional Chinese medicine (TCM) have been playing a certain role in fighting against coronavirus and influenza virus infection (49–51). The registered veterinary medicine Shegandilong preparation combined with doxycycline has been proved to prevent IBV infection and respiratory tract injury in broilers (52). The traditional Chinese medicine *Houttuynia cordata* (*Saururaceae*) had more than 90% inhibition rate against IBV infection in cells and more than 50% protection rate in SPF chickens against IBV challenge (53). Moreover, the classical prescription Qingwen Baidu Decoction is usually used to treat a variety of viral infectious disease, including the COVID-19 and IBV with a positive therapeutic impact (54, 55), though the decoction has not been officially approved for the treatment of COVID-19. Gancao Granules (甘草颗粒, in Chinese), Antitussive Power (止咳散, in Chinese), Banqing Granules (板青颗粒, in Chinese), Jinhua Antiasthmatic Power (金花平喘散, in Chinese), Panting-Stabilizing Power (定喘散, in Chinese), Yinqiao Power (银翘散, in Chinese) and many other TCM preparations recoded in Veterinary Pharmacopoeia of the People’s Republic of China can be used to prevent and cure the IBV infection. TCM preparations serve a crucial role in reducing economic loss for poultry farmers who do not vaccinate their chickens, who do not carefully execute biosafety precautions, and who do not strictly follow disinfection protocols.

## 4. Perspectives and recommendations for a better control strategy against IBV

There is no denying that TCM has played a huge role in fighting IBV infection in some poultry farms in China. However, for the future highly intensive, industrialized, automated and intelligent large-scale chicken farms, vaccine immunization is still the future trend. In the face of complex and changeable epidemic strains of IBV, what we need to do is to develop a better vaccine, implement a more effective vaccination strategy, and formulate a more optimized immunization program.

### 4.1. More better vaccines for IBV

Currently, there is one important problem for the IBV classical live and inactivated vaccines in China. The problem is that the research and development speed of novel classical vaccines cannot catch up with IBV virus mutation speed. With the gradual increase of variant strains and new serotypes of IBV, classical vaccines can no longer provide adequate immune protection for chickens against IBV infection. To battle against IBV infection, the research and development of novel vaccines of IBV is imminent. For novel IBV vaccines, it should not only avoid genetic recombination with field virus strains, but also provide enough protection for epidemic strains. Next generation vaccines should be able to combat future pandemic viruses and improve long-term protection against IBV variants. Messager RNA (mRNA) vaccine, viral vector vaccine and subunit vaccine against IBV infection might be the future research direction.

The COVID-19 epidemic has brought mRNA vaccine into the attention of people all over the world. Until November 15, 2022, WHO has reported 371 COVID-19 vaccine candidates in development, of which 65% are mRNA vaccines, and 40% of them have entered clinical trials, indicating that the mRNA has a potential future in preventing IBV infection.^2^ Compared with classical vaccines such as live and inactivated vaccines, mRNA vaccine has many advantages, including short development cycle, easy industrialization, simple production process, flexibility to respond to new variants, and the capacity to induce better immune responses (56). Brenner and colleagues reported the mRNA molecules for the first time in 1961 (57), and due to the highly unstable nature of the mRNA molecule and easy degradation, it was not until 1984 that SP6 RNA polymerase was employed for the first time to successfully transcribe and synthesize mRNA *in vitro*, laying the groundwork for further *in vitro* mRNA research (58). In 1990, *in vitro* transcription mRNA was successfully expressed after being injected into mouse muscle for the first time (59). However, mRNA vaccines have been widely disregarded due to the unsatisfactory stability and safety. This situation did not change until new uridine nucleoside modification was applied to synthesize mRNA to improve mRNA stability and immunogenicity *in vivo* in 2005 (60). In 2018, patisiran, the first-ever RNA interference therapy medication, was licensed and used to treat polyneuropathy of hereditary transthyretin-mediated amyloidosis in adults (61). Until the beginning of the COVID-19 pandemic in 2019, mRNA vaccines became a current research hotspot and the mRNA vaccines Pfizer-BioNTech (BNT162b2) and Moderna (mRNA-1,273) obtained emergency use authorization from many countries and regions, such as United States, the United Kingdom and some others (56). Nevertheless, there are still many problems to be solved before the advent of IBV mRNA vaccine. At present, the biggest problem is the cold chain storage of mRNA vaccines. The two most widely used mRNA vaccines in the world must be stored and transported in extraordinarily demanding ultra-low temperature environment from bottling to the moment before vaccination. The Pfizer/BioNTech vaccine needs to be stored at a very low temperature from −80°C to −60°C, and can only be stable for 2 hours after being thawed at room temperature; the modern vaccine is slightly stable, but it still needs to be stored at −20°C (62). Strict storage conditions limit the long-distance transportation of the vaccine, greatly increasing the transportation and storage costs of the vaccine, virtually pushing up the price of the vaccine itself, and finally leading to difficulties on popularizing the vaccine in developing countries. More new formulations have been designed to ensure stability of mRNA vaccine, such as the selection of excipients, formulation milieu, and manufacturing processes. Some lyophilized mRNA vaccines can break through the technical difficulties in the storage and transportation of mRNA vaccines (63). Therefore, mRNA vaccine for animals (especially for avian coronavirus) is feasible and worth expectation.

Viral vector vaccine against IBV is most likely to be commercialized and put into use in the poultry farm first compared with mRNA vaccine and subunit vaccine. There are examples of successful viral vector vaccines for both humans and animals. In China, registered commercial viral vector vaccines for poultry contain Bursal Disease-Marek’s Disease live Marek’s Disease vector vaccine and Marek’s Disease and Infectious Bursal Disease HVT vector vaccine. Inactivated recombinant baculovirus vaccine is the only viral vector vaccine for livestock, including Porcine Circovirus Type 2, Mink Enteritis Parvovirus and Rabbit Hemorrhagic Disease Virus. Moreover, the greatest benefit of viral vector vaccine is that a single vaccine may protect two infectious illnesses and simultaneously induce humoral and cellular immunity, whereas neither of the other two vaccines has this capability. Many types of virus vectors are available, and several types of virus vectors for poultry, such as Newcastle disease virus (NDV), fowl adenovirus (FAdV), herpesvirus of turkey (HVT), have been reported. Each virus vector vaccine has its own advantage. Among NDV, FAdV and HVT virus vector, NDV might be the best viral vector for IBV in three aspects. Firstly, IBV and NDV are vaccinated at the same time in most cases in poultry farm, that is why all registered IBV combined vaccines contain NDV vaccine, of which most are the LaSota strain (Table 2). Compared with FAdV and HVT virus vector, the NDV vaccine is widely used in poultry farm and the NDV virus vector is suitable for simultaneous immunization of NDV and IBV. Secondly, the avirulent NDV LaSota strain has long track records of safety and efficacy as a vaccine vector and can induce strong local and systemic immune responses against foreign antigens. Thirdly, based on the reverse genetic technology platform of NDV, the viral vector vaccine against the current epidemic IBV strains can be rapidly developed and can avoid the shortcomings of the gene recombination between attenuated virus vaccine and filed strain. The recombinant NDV vector vaccine expressing IBV antigen will be appreciated on chicken farms from the perspectives of optimizing the vaccine immunization program, safety and efficacy, and the timeliness of vaccine research.

At the present, the only genetic engineering subunit vaccine for poultry is infectious bursal disease virus subunit vaccine in China. Several registered subunit vaccinations are available for pigs, including swine fever, porcine circovirus type 2, streptococcus and *haemophilus parasuis* combination vaccine. Moreover, there is a recombinant subunit vaccination for sheep against *Chlymidia psittaci*. Overall, many difficulties need to be overcome to develop IBV subunit vaccine. A sustained and stable immune response cannot be produced by the purified pathogen protein generated *in vitro* due to the difficulty in maintaining stable protein structural epitopes. The immune response typically only affects humoral immunity and is unable to activate cellular immunity. Consequently, the number of doses of subunit vaccination increases, which indirectly raises the labor expenses of chicken farms. Moreover, the production method of protein extraction and purification directly raises the production cost of subunit vaccination. Therefore, the subunit vaccine, which is arduous and time-consuming, is not the optimal method for generating a novel IBV vaccine.

### 4.2. New vaccination route

Respiratory mucosal immunity plays an important role in preventing respiratory diseases, especially coronavirus infection. An aerosolized adenovirus type-5 vector-based COVID-19 vaccine (Ad5-nCoV) has been evaluated in a clinical trial and displayed good neutralizing antibody responses (64). Meanwhile, many poultry farmers immunize combined Newcastle Disease and Infectious Bronchitis live Vaccine by aerosolized inhalation at day-old. Compared with the routine vaccination route, spray immunization has many advantages, including extremely low labor intensity, and high mucosal immunity. Mucosal immunity can induce high secretory immunoglobulin IgA (sIgA), which is an important barrier of anti-infection immunity and exerts strong effects on preventing the adhesion and invasion of pathogens on the mucosal surface, dissolve bacteria, and neutralize viruses and toxins (65). Spray apparatus, droplet size, and vaccine adjuvant are the three main components impacting spray immunity, and the next step in the research area is to determine how to improve these three characteristics (66).

Chickens, especially for broiler/breeding breeders and laying hen, are intensively vaccinated with an average of 12–20 vaccine administrations per bird done within a production cycle (67). To reduce stress caused by vaccine immunization and avoid interference of maternal antibody, *in ovo* vaccination may be available immunization route. *In ovo* vaccination is performed at 18 day-old embryonated egg in broiler eggs coinciding with transfer of eggs from setter to hatcher compartment and it is adapted as a common practice worldwide now. Unlike coarse spray or water vaccination, *in ovo* vaccination ensures that each chicken can be immunized with precise, homogeneous distribution of vaccinations. More importantly, *in ovo* vaccination can induce early immune responses of the day-old chickens against circulating pathogens than post-hatch vaccination does. Therefore, *in ovo* vaccine is available against IBV combined with NDV, though there are some problems to be considered, including the virulence of vaccine strains on eggs, potential maternal antibody interference and immunosuppression observed with some vaccines (67).

### 4.3. More optimized immunization program

For the prevention and management of IBV, a rational and scientific immunization regimen is crucial. When designing an immunization program for a chicken farm, at least the following several elements should be considered. Firstly, pay attention to the prevalence and severity of local IBV. Then, assess the maternal antibody and immune responses induced by immunized vaccines. Moreover, choose the right available IBV vaccine and make sure the vaccination route and frequency. Finally, consider the effects of IBV vaccine on other vaccines and animal health.

There are numerous serotypes of IBV, and a single serotype of IBV vaccination cannot give sufficient protection against a heterologous challenge. Therefore, farmers use many vaccine serotypes to expand the scope of protection in their flocks, a practice known as “multi-monovalent.” (66). This strategy has been applied in poultry farms throughout the world due to its effectiveness in practice and principle. With the increasing emergence of novel serotypes and variants of IBV, the Mass and 793/B group IBV serotypes have been proved to be the widely used vaccine types and the largest breadth of cross-protection (68). In China, there are lots of Mass type vaccines and the commercial IBV vaccine of 793/B type has not been approved for clinical use until the IBV NNA vaccine filled the market gap of 4/91-like vaccines in 2018, which offer the farmers an opportunity to use this strategy.

## 5. Conclusions

Such a large scale of poultry in stock in China provides convenience for IBV gene mutation and recombination. IBV infection is still an important avian disease. Vaccine inoculation has replaced TCM therapy as the primary treatment method for chicken farms in addition to the stringent biosecurity measures. The optimal vaccine strains should be able to stop the spread of IBV variations and recombinants in addition to providing adequate protection against the classical IBV strains. The QX-like and 4/91-like strains are the main epidemic strains in China at the present. Based on the production cost, immunized route and efficacy of vaccine, the recombinant NDV virus vector vaccine expressed S gene of IBV QX-like and 4/91 strains will be the dominant vaccine strains against NDV and IBV.

## Author contributions

HL and CO conceived and designed this review. GeL, QZ, HY, and CZ participated in data analysis work. WK, JS, GoL, and HS performed the writing of the manuscript. WK and CO participated in revising the manuscript critically. All authors contributed to the article and approved the submitted version.

## Funding

This work was supported by National Natural Science Found (32260876), Guangxi Science and Technology Base and Talent Special (2021AC19372) and scientific research startup funds of Guangxi University.

## Conflict of interest

The authors declare that the research was conducted in the absence of any commercial or financial relationships that could be construed as a potential conflict of interest.

## Publisher’s note

All claims expressed in this article are solely those of the authors and do not necessarily represent those of their affiliated organizations, or those of the publisher, the editors and the reviewers. Any product that may be evaluated in this article, or claim that may be made by its manufacturer, is not guaranteed or endorsed by the publisher.

## Footnotes

^1^http://vdts.ivdc.org.cn:8081/cx/

^2^https://www.who.int/publications/m/item/draft-landscape-of-covid-19-candidate-vaccines
